# The Chemistry and Physiological Action of Mercury as Used in Amalgam Filling

**Published:** 1882-02

**Authors:** E. S. Talbot

**Affiliations:** Chicago.


AMERICAN JOURNAL

OF

DENTAL SCIENCE.

Vol. XV. THIRD SERIES—FEBRUARY, 1882. No. 10.

### ARTICLE I.

*The Chemistry and Physiological Action of Mercury as Used in Amalgam Filling.*

BY E. S. TALBOT, M. D., D. D. S., OF CHICAGO.

[Read before the Illinois State Dental Society. held at Rock Island, May 10th, 1881.]

The name Mercury was given by the ancients in honor of Mercurius, the messenger of the gods, whose volatile character mercury is supposed to typify. It is seldom found in the native state, but is usually combined. The most important as well as the most abundant combination is the sulphide of mercury, or cinnabar. It is found united with silver, forming an amalgam. The largest and richest mines are found in California. The process of obtaining pure mercury from the sulphide is very simple. The ore is mixed with one-half its weight of lime, and then distilled in iron retorts. The mercury is extracted and the lime remains in the retort. It is a heavy fluid metal, odorless, tasteless, of a whitish color, and free from other metals it does not tarnish, and its globules roll freely over white paper without leaving a streak or losing their form. It is liquid at ordinary temperatures. It boils at 662° F., and solidifies at—40° F. into a malleable mass of octahedral crystals. It is volatile at all temperatures, evaporation being much accelerated by the application of heat. The symbol is Hg, atomic weight 200. Mercury combines with other elements and radicals in two proportions. Those compounds in which the *lesser* acidulous radicals are united are termed mercurious. The higher mercuric—thus, calomel (HgCl) is mercurious chloride; while corrosive sublimate ($HgCl_2$) is mercuric chloride. Mercury combines with chlorine, iodine, bromide, lead, oxygen, phosphorus, sulphur, arsenic, bismuth, etc., forming compounds, some of which are used medicinally. The metal itself, rubbed up in confection of roses, licorice or suet until globules are not visible to the unaided eye, is used in medicine.

Amalgams were introduced into this country as fillings for decayed teeth in 1833. Since that time unlimited discussion has arisen among the general practitioners of medicine as well as the dentists, in regard to the practicability of utilizing this material in dentistry. The first amalgams were composed of pure silver and mercury, manufactured by M. Taveau. Later, Dr. Evans combined pure tin with a small quanity of cadmium, and Dr. Townsend formed an amalagam with four parts of silver to five parts of tin. Following these compositions came numerous others made from gold, silver, platinum and tin, until to-day we have more than a hundred varieties in the market, varying slightly in metals and proportions, that each manufacturer may rightly claim an orginal preparation. These different metals are melted in a crucible in their proper proportions by weignt, and poured into ingots. These are filled or cut into minute particles, ready for use. When the cavity in the tooth is prepared, a sufficient quantity of filling is rubbed up with mercury into a paste, the surplus mercury squeezed out, and the filling is inserted into the tooth.

From its first application as a filling the better class of dental practitioners waged war against it on general principles; not alone on account of the deleterious effects of the mercury in its composition, but because of its unsightly appearance and demoralizing effects upon the dental profession. The manner in which it was introduced into the country called forth the censure of all having a regard for professional etiquette: "Two adventurers, without skill or any claim to the title of dentist, suddenly appeared in New York and began dental practice amid such a shower of advertisements, a profusion of display, and a metaphorical flourish of trumpets, as caused our staid and dignified dental ancestry to bound with surprise and indignation."

From that time onward the use of amalgam has increased, until now tons are consumed yearly in filling teeth. Dr. Harris, in his opening address to the first class of the Baltimore College of Dental Surgery, in 1840, says: "It is one of the most objectionable articles for filling teeth that can be employed, and yet from the wonderful virtues ascribed to this pernicious compound by those who used it, thousands were induced to try its efficacy."

At the meetings of the dental societies this subject was spiritedly discussed with strong arguments against its use. The first official act in the matter was the appointment in 1841 of a committee by the American Society of Dental Surgeons to report on the use of lithodeon—mineral paste, and all other substances of which mercury is an ingredient for stopping teeth. They reported in substance that the use of all such articles was hurtful to the teeth and every part of the mouth, and that there was no tooth in which caries in it could be arrested and the organ rendered serviceable by being filled, in which gold could not be employed. This report was unanimously adopted.

At a meeting of the same society July 20, 1843, the use of amalgams was declared to be malpractice, and a committee appointed to further investigate the subject. They referred the matter to the Medical Society of the county of Onondaga, New York. The report of the medical committee was to the effect that no care in the combination or use of the paste will prevent its occasional bad effects.

In 1845, the Mississippi Valley Association of Dental Surgeons resolved that the use of Amalgam fillings was unprofessional and injurious, and would not be countenanced by its members. The actions of the various societies had very little effect; amalgam forced its way into the offices of the majority of dentists in the country. Many excellent practitioners were expelled, and others resigned from the societies to which they belonged.

In 1850, a resolution was passed unanimously by the American Society of Dental Surgeons to rescind the pledge made by the same society previously. Thus ended the so-called amalgam war. It will be observed that no scientific researches were made to ascertain whether deleterious effects were produced by mercury; the chief object of the disturbance was, apparently, to rid the profession of charlatans and their obnoxious materials. These discussions, which have caused so much bitterness and enmity among the members of societies, have latterly aroused a feeling of enquiry into the scientific analysis of this filling for teeth, resulting in the discovery of a quality in its composition capable of producing salivation, and all other symptoms of poisoning. Many able practitioners of dentistry have experimented with all the different acids with no satisfactory results. When we consider that nitric acid dissolves at 60° F.; concentrated sulphuric acid dissolves mercury only when heated; hydro-chloric acid does not affect it at all—how can we expect the weaker acids of the mouth, diluted by saliva, to cause a chemical change?

If passed experiments have proved unsatisfactory, it does not discourage me in the attempt to discover, if possible, by experiment and by the careful study of the subject of mercury in its every particular, some clue to this perplexing question. Having satisfied myself that the poisonous effects are not produced by the chemical changes in the mouth, I have entirely ignored this theory, and have looked about for a more simple and direct cause. In commenting upon mercurial poisoning, the idea has been advanced that the vaporization of mercury takes place only during the hardening process, and, that being consummated so quickly, no deleterious effects can occur.

In the construction of an amalgam two changes take place, the first being the mechanical, or mixing together of the ingredients, the second the chemical, or hardening of the composition. My first experiment was to place metallic mercury in a four ounce glass-stoppered bottle, and submit it to the gold test, by suspending a piece of gold foil in the centre of the bottle, taking care that it did not come in contact with the sides, and cementing the stopper. I placed it under different temperatures, of from 20 to 130° F. In about thirty-six hours the surface of the gold became coated with mercury, giving it a gray color. This proved the evaporation of the metal. I then mixed a quantity of the Chicago Refining Co.'s amalgam, according to their formula, three parts of mercury to eight parts of fillings, and subjected it to the same test. The reagent was not sufficiently delicate to produce any perceptible change. Wishing to bring the highest chemical skill to bear upon the experiment, I consulted Prof. Haines, of Rush Medical College, who kindly assisted me. At his suggestion, I procured two delicate reagents, the ammonio-nitrate of silver, and the chloride of platinum. In preparing the ammonio-nitrate of silver, I allow thirty grains of nitrate of silver to the ounce of water, and put a small quantity of the solution in the test tube. This was heated, and aqua ammonia added until a precipitate formed. Increasing the aqua ammonia, until the precipitate cleared up, I took a quill, and, with this liquid, wrote upon white paper. After putting the substance to be tested in the bottle, the strip of paper was placed across the mouth of the bottle, and the stopper cemented. Should a vapor arise, the liquid would become black. Leaving the bottle for ten minutes, I examined it again, and found the writing in plain black coloring. The chloride of platinum produced the same results, but required more time to accomplish it. The rapidity with which the evaporation of mercury takes place depends upon the amount of heat and the surface exposed, and not upon the quantity of mercury contained in the fillings. Thus a jar containing one quart of water would evaporate the same quantity as a jar of like surface containing a gallon, the latter taking four times longer to empty.

In the following experiments, I attached a thermometer to a water bath, and heated to the temperature of the body, 98° to 100° F., to maintain an even temperature. I conducted these experiments in the dark, as the rays of light decompose the ammonio-nitrate of silver. The strips of paper containing the reagent were placed in the mouths of all the bottles, including an empty bottle, which was used in each experiment, to prove there was no mistake.

Experiment No. 2.—Three bottles were prepared. In the first was placed an amalgam filling made from Chicago Refining Company's amalgam, according to their formula. In the second was placed an amalgam filling of like size, containing five grains more of mercury. In the third bottle there was nothing. After a lapse of ten minutes I examined the bottles and found the writing on the paper across the mouth of bottles Nos. 1 and 2 was black, while there was no discoloration of paper in the third bottle.

Experiment No. 3.—A repetition of No. 2, with the exception of the reagent chloride of platinum being substituted for the ammonio-nitrate of silver. The results were the same in both. The time required for the latter being ten hours, while but ten minutes were required for the ammonio-nitrate of silver. In conducting the remainder of the experiments the ammonio-nitrate of silver was employed, it being the more delicate reagent, consequently producing a more marked impression, and also consuming less time than chloride of platinum.

Experiment No. 4.—Two bottles were prepared. In the first bottle were placed scraps of amalgam six months old. In the second there was nothing. In ten minutes the writing on the paper was black in the first bottle, and uncolored in the second.

Experiment No. 5.—Bottle No. 1 contained amalgam fillings which had remained in the teeth from two to ten years. Bottle No. 2 was empty. The results in both being the same as in experiment No. 4.

Experiment No. 6.—In bottle No. 1 I put an amalgam filling which had been in the mouth sixteen years. In bottle No. 2 there was nothing. At the end of twenty-four hours I found the paper discolored in the first, and not in the second.

Experiment No. 7.—To demonstrate that nothing in the composition of the fresh fillings could cause the discoloration I allowed some fillings to remain sealed in the bottle for some twenty-four hours. At the end of that time discovered no signs of color on the paper.

Experiment No. 8.—I procured four preparations of mercurious vivus.

| | | | | | | |
|---|---|---|---|---|---|---|
| No. 1 | contained | 1-10th | gr. mercury | to 1 gr. | of sugar | of milk. |
| No. 2 | " | 1-100th | " | " | " | " |
| No. 3 | " | 1-1000th | " | " | " | " |
| No. 6 | " | 1-1000000th | " | " | " | " |

A small quantity of each of these preparations was placed in bottles marked 1, 2, 3, 4. In No. 5 there was nothing save the reagent. The effect was alike in each of the four bottles containing the mixture. Those ha ing the greatest quantity of mercury caused the deepest color to the paper and required less time. As before, No. 5 was unaffected.

In order to determine the difference, if any, in weight after evaporation, I obtained strong glass tubes one-half inch in length, and one fourth inch in diameter, and packed them carefully with amalgam fillings. Allowing twenty-four hours for hardening, I weighed them, and at the end of three months 1 again weighed them, finding in some no change at all, and in others an increase of weight. This is accounted for by the fact that oxydation and accumulation of moisture on the amalgam equaled in some and exceeded in others the loss of weight by evaporation. I am in possession of numberless cases of poisoning from mercury in amalgam fillings. I will mention but one, and report one case from my practice.

The *Dental Register*, January, 1872, has the following case of poisoning from mercury in a tooth filling: "John T. Smith died from salivation, caused from having a tooth filled with amalgam. Dr. Sprague attended the case, and afterwards called Drs. Davis and Buffin, all of whom agreed that he was suffering from the effects of mercury present in the amalgam used in filling one of his teeth. The filling had salivated the unfortunate man, and, as the inside of his mouth, throat and windpipe swelled, respiration was hindered, and finally ceased altogether. Dr. Davis made the post-mortem examination in the presence of the coroner and jury of inquest, opening the chest, taking out the lungs, and extracting the filled tooth. No signs of any other diseases were found, except that caused by the mercury, and it was made clear to the jury by the Doctor that this caused his death. The jury returned a verdict that the deceased came to his death by suffocation, caused by inflammation of the glands and filtration of the tissues of the neck, producing closing of the træchea by pressure thereon; 'and we further believe that the above causes were brought about by the action of mereury, used in filling the second molar tooth of the right side of the lower jaw, by Dr. E. D. Keef, Marysville, Kansas.'"

A case in practice: A lady from one of the towns in Illinois came to Chicago for treatment, having been troubled with dyspepsia and nervous debility for two years. While under the physician's care she complained constantly of a peculiar feeling and taste in her mouth. The doctor suspected the trouble might arise from a rubber plate which she had worn for four years, and advised her to consult me. Upon examination, I found a full upper plate, composed of rubber, and on the lower jaw the molars were gone, except the second and third upon the left side. In the crown of the wisdom tooth was a large amalgam filling, and also one in the crown and posterior approximal surface extending to the free margin of the gum in the second molar. These had been inserted about two years previous. I noticed that the gums and the mucous lining of the mouth and salivary glands were quite tender. There was a strong metallic taste in the mouth, and a metallic odor to the breath. She had a peculiar paralyzed sensation in the left side of the tongue, which she had experienced for two years. She also informed me that the saliva flowed so freely that at night her clothing and pillow were saturated, and estimated the loss of saliva each night to equal one pint. I suggested the removal of the amalgam fillings and rubber plate, and substitution of gold. She assented to the proposition, and as easily as possible I undertook the operations. Upon removing the amalgam fillings and applying the rubber dam, the saliva flowed in streams, completely saturating several towels. After refilling the teeth, and inserting a gold plate, the unpleasant sensation in the tongue and metallic taste disappeared. At the end of two weeks the glands were greatly improved, and the soreness under the tongue (of which she had complained at her first visit) was healed.

It is the accepted opinion of physicians generally, that mercury uncombined has no constitutional effect. Dr. Atkinson said before the meeting of the American Dental Association in Boston, August. 1880: "You must combine the molecules of mercury with some other agent before they can have any affinity for the body at all. One who is familiar with the old method of making looking-glasses, with tin foil and mercury, knows that the workman would be literally saturated with it, so that he could not be capable of handling a gold or silver watch without its becoming amalgamated, and all this, too, without his health being compromised by the mercury so long as it remains in a metallic state."

The correctness of this theory may be questioned, as it has been proven that these workmen have been affected by the vapor of mercury, when not protected by a veil over the mouth and nose.

Dr. Bartholow, in his work on Therapeutics, p. 177, says: "As used in the mechanical arts, by gilders and others, the fumes of mercury cause wasting, ptyalism, necrosis of bones, trembling, impaired intellect, and, in women, abortion." "Walter Pope mentions a workman who for six months had not handled mercury; yet he rendered a piece of copper as white as silver by rubbing it between his fingers." Parish says that long trituration of calomel increases its power to salivate. This is also applicable to all preparations of mercury used with an incipient, medicinally. The homeopaths divide and subdivide particles, according to the required preparations, some of the radical members of the school claiming best results from the highest potencies, while the more conservative practitioners prefer a middle ground. They rub up pure mercury with sugar of milk into six different grades, the first containing one-tenth gr. of mercury to one gr. sugar of milk; the second, one one-hundredth gr. mercury to one gr. sugar of milk, etc., as before mentioned in this paper. These are the finest forms in which mercury is prescribed, and yet the severest cases of salivation and constitutional symptoms have been produced by these agents, on account of their being so readily taken up by the blood. Is it not a reasonable supposition that, if poisonous symptoms are produced in proportion with the subdivision of the particles of mercury, that the system will be more severely affected by the vapor of mercury, which is finer than any mechanical subdivision can be? Dr. Somers recalls an instance of a lady patient becoming completely salivated, the gums and mucous lining of the mouth inflamed and teeth loosened, by taking a second bath, in which forty grains of the black oxide of mercury were used. He thinks she could not have absorbed one-twentieth of the amount in the form of vapor.

As a forcible illustration, I quote the experience of the sailors on board the man-of-war "Triumph," which, in April, 1810, took from the wreck of a Spanish ship thirty tons of quicksilver, contained in bags of fifty pounds each. In the course of a fortnight some of the bags decayed and burst, the quick-silver mixing with the bilge water, the emanations from which coated all the metal about the ship. Nearly all the crew were salivated.

In order to ascertain the effects of the vapor of mercury, I have employed it in a series of experiments upon plants and animals.

Experiment No. 1.—While conducting my experiments in the laboratory I was frequently visited by a family of roaches, who appeared to take an interest in my operations. Suddenly they all disappeared, and it immediately suggested itself to my mind that their sudden departure argued favorably in the question of utilizing them in my experiments. I took four two-ounce bottles and put in No. 1 pure mercury; No. 2, amalgam scraps six months old; No. 3, fillings from two to ten years old; No. 4, fillings sixteen years old. After placing a roach in each bottle, I tied a piece of cloth over the mouth in order that the air might circulate. Evidently the bugs were not fond of mercury, for they clung to the tops of the bottles as long as life last. Roach in No. 1, containing pure mercury, died in three days; roach in No. 2 was next to follow; roach in No. 3 lived a few days longer; and in No. 4 outlived them all by several days.

Experiment No. 2.—I prepared three bottles. The No. 1 contained ten grains of pure mercury; No. 2 contained an amalgam filling three months old; No. 3 was an empty bottle. In each of the bottles I put two roaches. In two days one in the bottle containing pure mercury died; the remaining one in the same bottle lived nine days from the time it was put in. In the bottle containingthe amalgam filling one roach died in four days, while the other one died in eleven days; while those in the empty bottle lived fifteen and sixteen days.

Experiment No. 3—On February 9th I placed an amalgam filling at the base of a sensitive plant. On examination, about the fourth day, I discovered that the extremities of the leaves had changed color and were dry and brittle, like the leaves in early Autumn; gradually the whole leaf was affected, and at the end of ten days the plant was dead, notwithstanding the care and nourishment it received.

Experiment No. 4.—In a four-quart glass jar I put about four ounces of mercury, and made a platform of wire gauze, fastening it two inches from the bottom of the jar. I placed a guinea pig in the jar, and covered the top with gauze. Twice each day I removed him for exercise and nourishment. He thrived well for ten days, but at the end of that time he commenced to droop, and refused food and water. He became emaciated and trembling; the body and limbs were cold. He lingered along for two weeks and died.

Experiment No. 5.—I administered six grains of mercurius vivus, first trituration, to a dog with his supper, and repeated the dose next morning with his breakfast. The blood and liver were examined under the microscope in the evening and found to contain globules of mercury.

It is the opinion of many eminent scientists that mercury inhaled into the lungs produces a greater effect than when taken into the stomach. Among this number Prof. Stille in his Therapeutics, Vol. 2, page 789, says: "Of the several modes by which mercury is made to enter the body, inhalation most speedily produces the specific influence of the medicine." Blaude Bernard, late Professor in "Le College de France," makes the same statement in one of his lectures. This is readily understood when we consider that the drug taken into the lungs in the form of vapor is distributed over a large surface and brought in direct contact with oxygenated blood, and thus carried directly to all parts of the body.

Mercury taken into the system in small quantities, long continued, manifests itself in a variety of ways. One of the first symptoms noticeable is an increased flow of the secretions of the body—salivation being the most striking—the glands becoming inflamed and the mucous membrane tender. The gums tumefy and change in color to a dark rose tint; the tongue is swollen; the patient not only experiences an unpleasant metallic taste, but the breath becomes impregnated also. Sometimes extensive ulcers attack the throat, gums and cheeks; œdema of the glottis, with difficulty in breathing and swallowing. The digestive apparatus is involved, with loss of appetite, nausea and vomiting, and frequently pain and tenderness of the stomach; the bowels loose, and often bloody stools. The fatty constituents are removed, and the patient becomes emaciated; no part of the body is more affected by mercury than the nervous system; the body trembles; sometimes one limb, and again both limbs contracted. A sense of coldness and occasional chills are experienced; often neuralgic pains are felt, particularly around the motor nerves; mental debility and loss of memory. These are some of the many symptoms caused by the inhalation of the vapor of mercury.

**RESUME.**

There are in the market many varieties of amalgams. Evaporation does not depend upon quality or age, but all amalgams will send off the vapor of mercury. This has been proved conclusively by its destruction of animal and vegetable life, and by chemical tests. Evaporation is facilitated by an increase of surface, consequently a greater amount of vapor would arise from several small fillings than from one large filling. The facility with which mercury is taken into the lungs by continued inhalations and the rapidity with which it enters the blood, requires less mercury to produce systemic effects then whan taken into the stomach. In order to produce systemic effects from metallic mercury, it must be rubbed up with an excipient, to reduce the particles to a size capable of entering the capillary system, or it must be taken into the lungs in the form of vapor.—*Ohio Jour. of Dent. Science.*

